# Human Resource Information System implementation readiness in the Ethiopian health sector: a cross-sectional study

**DOI:** 10.1186/s12960-017-0259-3

**Published:** 2017-12-20

**Authors:** Eyilachew Dilu, Measho Gebreslassie, Mihiretu Kebede

**Affiliations:** 1 Department of Health Service Management, College of Medicine and Health Sciences, University of Gondar, Gondar, Ethiopia; 2 Department of Health Informatics, Institute of Public Health, College of Medicine and Health Sciences, University of Gondar, P.O. Box 196, Gondar, Ethiopia; 3 Leibniz Institute for Prevention Research and Epidemiology - BIPS , Achterstrasse 30, Bremen, 28359 Germany

**Keywords:** HRIS, E-HRM, Electronic Human Resource Management, Readiness, Human Resource Information System, Ethiopia

## Abstract

**Background:**

Health workforce information systems in low-income countries tend to be defective with poor relationship to information sources. Human Resource Information System (HRIS) is currently in a pilot implementation phase in the Federal Ministry of Health and Regional Health Bureaus of Ethiopia. Before scaling up the implementation, it is important to understand the implementation readiness of hospitals and health departments. The aims of this study were to assess the readiness for HRIS implementation, identify associated factors, and explore the implementation challenges in public hospitals and health departments of the Amhara National Regional State, Ethiopia.

**Methods:**

An institution-based cross-sectional study supplemented with a qualitative study was conducted from the 15th of February to the 30th of March 2016 in 19 public hospitals and health departments of the Amhara National Regional State, Ethiopia. A self-administered questionnaire was used to collect the data. The questionnaire includes items on socio-demographic characteristics and questions measuring technical, personal, and organizational factors adapted from the 32-item questionnaire of the Management Science for Health (MSH) HRIS readiness assessment tool. The data were entered and analyzed with statistical software. Descriptive statistics and bivariate and multivariable logistic regression analyses were performed. Odds ratios with 95% confidence interval were computed to identify the factors statistically associated with readiness of HRIS implementation. In-depth interviews and observation checklists were used to collect qualitative data. Thematic content analysis was used to analyze the qualitative data.

**Result:**

A total of 246 human resource (HR) employees and 16 key informants have been included in the study. The HR employee’s level of readiness for HRIS implementation in this study was 35.8%. Employee’s Internet access (AOR = 2.59, 95%CI = 1.19, 5.62), availability of separate HR section (AOR = 8.08, 95%CI = 3.69, 17.70), basic computer skills (AOR = 6.74, 95%CI = 2.75, 16.56), and fear of unemployment (AOR = 2.83, 95%CI = 1.27, 6.32) were associated with readiness of HRIS implementation. Poor logistic supply, lack of competency, poor commitment, and shortage of finance were the challenges of HRIS implementation.

**Conclusion:**

In this study, readiness of HRIS implementation was low. Strategies targeting to improve skills, awareness, and attitude of HR employees would facilitate the implementation process.

**Electronic supplementary material:**

The online version of this article (10.1186/s12960-017-0259-3) contains supplementary material, which is available to authorized users.

## Background

Efficient and effective management of human capital is increasingly becoming very important. As a result, there has been a considerable increase in the number of health institutions gathering, storing, and analyzing information regarding their Human Resources (HR) through the use of Information and Communication Technology [1].

In 2010 WHO (World Health Organization) technical meeting to strengthen health workforce information systems in low-income countries, it was reported that the Human Resource Information System (HRIS) of the low-income countries tend to be defective with poor relationship to other information sources [2]. Poor management of HR for health data, low utilization of HRIS for health policy, and incompetency of employees in handling computerized information systems were the weaknesses reported from low-income countries [2, 3].

HRIS is an efficient and well-organized catalyst for connecting HR management and Information Technology [4]. It is a database system that is developed to provide the necessary support to human resource management (HRM) in terms of collecting and analyzing HR data, decision-making, and reporting of HR information [5, 6]. It is increasingly becoming an integral part of national HR for health performance assessment and a valuable tool for health systems strengthening [7, 8].

To improve the routine manual process, speed up the often slow HRM process, and deal with the transformational changes, implementing HRIS is crucial. The implementation of HRIS has positive impact on the performance of hospitals, health departments, and other healthcare organization [9–11]. However, its implementation in many low-income countries has been a challenging task. Challenges such as shortage of expertise, technical difficulties, shortage of finance, lack of technically skilled HR, and top management support failures and dedication have lagged the implementation process in many low-income countries [9, 12, 13]. In addition, deficient HR knowledge by system designers, lack of appliances for HR users, lack of competent HRIS employees, failure to work in teams with other departments, and failure of information technology support were identified as challenges [9, 12, 14, 15]. Due to such challenges, low-income countries such as Tanzania took more than 6 years to roll out HRIS and to make it a strong data source for human resource for health (HRH) and social welfare workers in Tanzania [12].

The global review of information systems on HR for health in 2013 reported that Ethiopia is one of the WHO designated HRH crisis country [3, 16]. In Ethiopia, HRIS mainly relies on paper-based system by which the personnel department had to manually collect data from applicants and employees in order to keep employees’ information [17]. Moreover, there is no HRIS and no proper mechanism to manage data about the existing health work force [18, 19]. Hence, the whole process of HR system is tremendously time-consuming, and the core HR processes (such as recruitment and selection) are liable to data discrepancies due to lack of reliable information system in place [17, 19, 20].

In Ethiopia, the main HR functions have been allocated under two separate arms of The Federal Ministry of Health (FMoH): Human Resource Development Directorate, and Health Professionals and Facilities Licensing and Regulation conducted by Food, Medicine and Health Care Administration and Control Authority (FMHACA). Projected estimates show that the HRH requirements in Ethiopia will increase by more than threefold in the coming decades. Therefore, Advanced HRIS is crucial and timely for the efficient management of HR system in Ethiopia [19, 21]. Because of this, in the new Health Sector Growth and Transformation Plan, the Ethiopian FMoH has planned to implement HRIS in health institutions aiming to revolutionize HRH core processes and facilitate health system strengthening [19, 22]. However, the implementation faces challenges and factors that hinder a successful implementation [19, 20, 23]. Due to these challenges, the currently being piloted HRIS is at risk of being abandoned [23].

According to literature, readiness of HRIS implementation is influenced by the demographic characteristics of employees, availability of skilled human power, organizational structure, and technological factors [10, 24, 25]. Evidence from Tanzania suggested that strong involvement of higher officials, reliable technical support for the system users, provision of training, and close follow-up were the key success factors for implementation of HRIS [12]. However, lack of supportive supervision, non-reliable internet connection, insufficient infrastructure, and pre-conceived negative experience of system users are also cited as challenges of implementation [12, 23].

Studying readiness of HRIS implementation is crucial to predict the HRIS success or failure. As system change often causes change resistance, successful change needs to have a common understanding of the objectives among all stakeholders, users, and implementers who have direct relationship with the HRIS implementation [26]. Managers and stakeholders need to use their positive influence to make individuals, groups, and organization exchange the same vision of change. Hence, factors associated with readiness and challenges limiting the implementation need to be studied before the implementation. Therefore, this study aimed to (1) investigate readiness of employees towards HRIS implementation, (2) identify factors associated with implementation readiness, and (3) explore challenges of HRIS implementation among HR employees of the public hospitals and health departments of the Amhara National Regional State.

## Methods

An institution-based cross-sectional study triangulated with qualitative study was conducted from the 15th of February to the 30th of March 2016 in Amhara National Regional State in public hospitals and health departments. Amhara National Regional State is one of the nine regional states in Ethiopia and inhabits the second largest population of the country. The source population for this study was HR employees, medical directors, managers of public hospitals, and heads of the zonal and town administration health departments. The study population consists of all HR employees, medical directors, managers, and vice mangers of 19 public hospitals and 10 zonal and 3 town administration health departments in the region. Respondents having more than 3 months of work experience were included in the study. Employees who were in their annual leave and/or sick leave during the data collection period were excluded from the study.

The sample size for this study was determined using a single population proportion formula. Where *N* = size of the study population, *n* = sample size, *p* = proportion of readiness to implement HRIS to 50%, *d* = desired level/margin of error (5%), *z* = standard normal distribution curve value for the 95% confidence interval (1.96). Based on the formula, the sample size was calculated to be 166 respondents. We included all the 288 HR employees. To explore the challenges of HRIS implementation, qualitative data were collected using semi-structured in-depth interview and an observation checklist. Following recommendations from qualitative study methodological guidelines [27, 28], we continued the in-depth interviews until level of saturation or to the point where no emergent opinion was reached. Hence, 16 of the 83 managers/vice managers of the hospitals and zonal health departments were included in the in-depth interview.

### Data collection procedure and tools

Data were collected from HR employees of health departments using self-administered questionnaires (Additional file 1). In-depth interview and observation checklists were used to collect qualitative data from key informants composed of heads of HR departments, managers, and medical directors of hospitals and zonal health departments. Readiness of HRIS implementation was assessed by adapting the 32-item Management Science for Health HRIS assessment tool [29]. The tool was preferred as it has been validated in developing countries context [15]. We also pretested the tool and found that it is applicable in the Ethiopian context. Factors that could potentially affect the organizational readiness of HRIS implementation were selected from the literature. Finally, an observation checklist was used to supplement the quantitative data. The contents of this checklist include items helpful to observe HR infrastructure such as electric power availability, availability of separate HR sections for HR core processes, fulfillment of the ergonomics of HR, availability of computer and its accessories, and availability of necessary office setups.

The self-administered questionnaire for collecting the quantitative data was first prepared in English, translated to Amharic, local language, and then translated back to English by language experts to check for consistency. The questionnaire which was used to collect data from all respondents was in the local language, Amharic. Ten trained Health Information Technicians (HIT) were assigned to collect the data. The entire data collection process was supervised by two data collection supervisors employed for the study.

### Data quality management

Questionnaires were given to the respondents after explaining the purpose of the study and encouraging them to provide genuine responses. Respondents were briefed concisely without losing the perspective of the intended objective of the questionnaire. In addition, the layout was designed to be simple to attract the respondents. A 2-day training on how the date should be collected was given to 10 HIT. Frequent supervisions on the data collection process to ensure the completeness and consistency of the gathered information and errors for example missing values and incomplete questionnaire found during the process were corrected.

### Data processing and analysis

The data were entered using EPI info version 3.5.1 software and analyzed using SPSS version 20 statistical package. Data cleaning was performed to check for frequencies, accuracy, and consistencies and missing values and variables. Errors identified were corrected through rechecking each respondent’s questionnaire. Frequencies, proportion, and summary statistics were used to describe the study population in relation to relevant variables. Bivariate and multivariable logistic regression analyses were carried out to investigate the effect of each independent variable on the dependent variable. Independent variables with *P* value of less than 0.2 were taken into multivariable logistic regression analysis to control the effect of confounding. Adjusted odds ratios with 95% confidence interval were then used to identify the factors associated with the readiness of HRIS implementation. The qualitative data were analyzed through thematic content analysis.

Readiness of HRIS Implementation was assessed by adapting the 32-item Management Sciences for Health HRIS readiness assessment tool. Those who score above the mean value of the 32 (yes/no)-item questions measuring the technical, organizational, and personal requirements were operationally defined as “ready” else “not ready.”

## Results

### Socio-demographic characteristics of the study population

A total of 246 human resource employees were included in the study, and the overall response rate was 85.4%. The respondents were working in 32 health organizations, i.e., in 19 public hospitals and 10 zonal and 3 town administrative health departments. Of the total study subjects, 131 (53.3%) were females. The mean age of the study subjects was 34.5 with an SD of 7.3 years, and 128 (52%) of them had first degree and above.

Among the HR employees, 103 (41.9%) of them had a monthly salary from 2351.00 (low) to 3350.00 (middle) Ethiopian birr (1 US$ = 23 Ethiopian birr). Regarding their work experience, 75 (30.5%) had less than 5 years of work experience (Table 1).

**Table 1:** Socio-demographic characteristics of HR employees in public hospitals and health departments, Amhara Regional state (*N* = 288)

Variables	Frequency	Percent
Age		
Below 25 years	26	10.6
26–30 years	64	26.0
31–35 years	57	23.2
36–40 years	58	23.6
Above 41 years	41	16.7
Sex		
Female	131	53.3
Male	115	46.7
Marital Status		
Single	71	28.9
Married	157	63.8
Divorce	16	6.5
Widowed	2	.8
Religion		
Orthodox	194	78.9
Muslim	30	12.2
Protestant	22	8.9
Educational Status		
College diploma and below	118	48.0
First degree and above	128	52.0
Position		
Data clerk	12	4.9
HR process owner/manager	18	7.3
HR case workers/employees	183	74.4
HIT and others	33	13.4
Enrollment		
Regular program	125	50.8
Distance learning	110	44.7
Upgrading/in-service training	8	3.3
Others	3	1.2
Work experience		
Below 5 years	75	30.5
6–10 years	72	29.3
11–15 years	49	19.9
16–20 years	34	13.8
21 + years	16	6.5
Monthly salary		
Below 2 350.00	61	24.8
2 351.00–3 350.00	103	41.9
Above 3 351.00	82	33.3

A total of 16 key informants participated in the in-depth interview of hospital managers, department heads, and medical directors. Of these, six were hospital managers, four health department heads, four HR department managers, and two medical directors.

### Overall readiness of HRIS implementation

Readiness was determined based on whether the value calculated from the total of 32 items analyzed was above or below the mean. Hence, the overall readiness to implement HRIS in Amhara Regional State public hospitals and health departments was 35.8%.

Regarding the infrastructure of the hospitals and health departments, 198 (80.5%) respondents had telephone access in their working organization for office work purpose, and 220 (89.4%) had electric power access. This was also confirmed by the observation made in the hospitals and departments. The majority of the respondents 193 (78.5%) had at least one to three computers in their HR department, one computer being shared among a maximum of 10 HR staffs; in most cases, only one computer is available for one HR core process.

The majority of the respondents, 220 (89.4%), had no personal computer for themselves. Internet and network access were not available for 197 (72.8%) respondents. Regarding their office structure, half of them, 122 (49.6%), had no separate room for HR section. This was also confirmed by the observation made but each of the respondents had their own seats to perform their daily tasks. Eighty-seven percent of the respondents replied that their HR section had no separate budget. Concerning information technology support, 230 (93.5%) reported that they did not have anyone in their organization being qualified to keep the computer(s) functioning and to deal with any malfunctions (Table 2).

**Table 2:** Organizational factors of the HR employees in public hospitals and health departments, Amhara Regional state (*N* = 288)

Variable	Frequency	Percentage
Telephone access		
Yes	198	80.5
No	48	19.5
Electric power		
Yes	220	89.4
No	26	10.6
Number of computer in HR section		
No computer	12	4.9
1–3 computers	193	78.5
4 and above computers	41	16.7
Personal computer		
Yes	26	10.6
No	220	89.4
Internet access		
Yes	67	27.2
No	179	72.8
Network access		
Yes	67	27.2
No	179	72.8
Functional printer access		
Yes	205	83.3
No	41	16.7
Backup power supply		
Yes	12	4.9
No	234	95.1
Separate HR section		
Yes	124	50.4
No	122	49.6
Separate budget allocation for HR section		
Yes	32	13.0
No	214	87.0
Information technology supporter		
Yes	16	6.5
No	230	93.5

A respondent at the zonal health department head said: “In zonal and town administration health departments, the HR section and the supply and logistic departments are organized together in one case team. Owing to this, separate budget and technology advancement is not as it was expected. There is no ICT (Information Communication Technology) support and we often have difficulties whenever we need support for computer maintenance and troubleshooting”.

A 36-year-old, male HR manager described the situation

“The HR section is well organized and based on the ‘Kaizen’ principles but each employee has not his/her own personal computer on his/her seats. The only employees who have their own computer on their table are the HR manager and the data clerk-secretaries.”

During the observation sessions of this study, most of hospitals’ and health department’s HR section offices were not well organized. It was observed that they had small and uncomfortable offices. In the observation of the 32 institutions, 17 (53%) had no separate room for the HR section. One HR employee in a hospital said: “HR section is simply seen by executives as a room where incoming and outgoing letters get registered.” He added that: “It needs the attention of executives and professionals with appropriate software training to make the human resource management a technologically supported work area/section.”

Of the total respondents, 173 (70.3%) of them did not think that there is a developed information technology infrastructure in their organization. The respondents were asked whether their computers will be suitable to use HRIS; 80 (32.5%) replied it is adequate. The respondents were also asked whether the HRIS will be user-friendly or not; 104 (42.3%) think that it will be user-friendly. In addition to that, 127 (51.6%) also expect that HRIS software will be compatible with the current HR workflow (Table 3).

**Table 3:** Technical factors of the HR employees in public hospitals and health departments, Amhara Regional state (*N* = 288)

Variables/question	Frequency	Percentage
Do you think that there is a developed information technology infrastructure (i.e., hardware, software, networks), and human expertise?		
Yes	73	29.7
No	173	70.3
Is your computer comfortable to use?		
Yes	80	32.5
No	166	67.5
Do you use anti-virus for your computer?		
Yes	89	36.2
No	157	63.8
Do you think that HRIS software will be user friendly?		
Yes	104	42.3
No	142	57.7
Do you think that HRIS software will be compatible with the HR workflow?		
Yes	127	51.6
No	119	48.4

A 39-year-old primary hospital manager said: “The HR section is separate and ergonomically is good but the infrastructure (internet, network, per-head personal computers etc.) are not well furnished. In addition to these, employees are not skilled to use computers unless they receive trainings”. A 32-year-old male HR manager of a referral hospital also described “HRIS is a very good system if employees are technically aware of how to run the software from recruitment until the exit of employees in the organization.”

Regarding the perceived knowledge about the HRIS, 154 (62.6%) reported they did not know the modules of HRIS, while 138 (43.9%) did not know the advantages of HRIS. When we analyze the respondents’ basic computer skills, 100 (40.7%) reported they had no skill at all. Regarding the ability to install and configure HRIS, 179 (72.8%) and 195 (79.3%) reported that they could not install neither configure the HRIS. In addition to this, 186 (75.6%) reported that they do not have the skills to manipulate the features of HRIS.

During the observation of HR employees in their practical work area, it was observed that the respondents’ lack basic compute knowledge and skills.

A 41-year-old male hospital manager described, “Most of the employees in human resource management have no basic computer skill and there are only two old version computers. They are not enough for eight HR employees in the section. The HIT who are employed before one year will have great contribution for the future to implement HRIS since they have a generic skill more than the HR employees having only basic skills.”

Regarding their perceived attitude about the importance of HRIS software, the majority of the respondents, 209 (85%), reported they believe HRIS are important. Majority, 207 (84.1%), of the respondents thought that they have a role in the implementation of HRIS in their organization. They were asked about their fear of HRIS creating unemployment; 86 (35%) respondents thought that it would make them lose their jobs.

A 41-year-old male HR manager mentioned that,

“Clearly, those HR employees need to have good perceived attitude about HRIS and it’s important for the effective and efficient work accomplishment of human resource management functions. On the contrary employees may be afraid that they will be fired if their work is to be replaced by computers and done by small number of employees.”

Concerning the respondents’ training on the HRIS utilization, 182 (74%) did not take any training. Of the total participants, 174 (70.7%) reported that there was no introduction of HRIS for all employees in their department. Furthermore, about their responsibility for the full implementation of HRIS; 178 (72.4%) of them answered that they felt responsible. The respondents were also asked whether HRIS will enhance efficiency of HRH for organizations. More than four fifths, 213 (86.6%), responded that it could help enhance HRH efficiency. Also, 185 (75.2%) of the respondents replied that they believed that the HRIS is applicable in their organization (Table 4).

**Table 4:** Personal factors of the HR employees in public hospitals and health departments, Amhara Regional state (*N* = 288)

Questions/variables	Frequency	Percentage
Do you know the modules of HRIS?		
Yes	92	37.4
No	154	62.6
Do you know Advantages of HRIS?		
Yes	108	43.9
No	138	56.1
Have you basic computer skill?		
Yes	146	59.3
No	100	40.7
Can you install HRIS software?		
Yes	67	27.2
No	179	72.8
Can you configure the HRIS software?		
Yes	51	20.7
No	195	79.3
Can you manipulate all the HRIS modules?		
Yes	60	24.4
No	186	75.6
Do you think that HRIS is important for your organization?		
Yes	209	85.0
No	37	15.0
Do you think that you have roles in implementation of HRIS?		
Yes	207	84.1
No	39	15.9
Did you receive training on HRIS?		
Yes	64	26.0
No	182	74.0
Is there a manual or handbook on HRIS?		
Yes	76	30.9
No	170	69.1
Is there a starting of the HRIS for all employees in your department?		
Yes	72	29.3
No	174	70.7
Do you think that HRIS will create unemployment?		
Yes	86	35.0
No	160	65.0
Do you feel that you are responsible for full implementation of HRIS?		
Yes	178	72.4
No	68	27.6
Can HRIS enhance efficiency of HRM in the organization?	213	86.6
Yes		
No	33	13.4
Can HRIS software be fully applicable in your organization?		
Yes	185	75.2
No	61	24.8

### Factors associated with readiness of HRIS implementation

Bivariate analysis shows that educational status, work experience, availability of internet access, availability of separate HR section, personal computer, availability of information technology support, basic computer skill, and fear of unemployment were significantly associated with readiness at a *P* value of less than 0.2.

However, the multivariable logistic regression analysis identified that internet access (AOR = 2.59, 95%CI [1.19, 5.62]), availability of separate HR section (AOR = 8.08, 95%CI [3.67–17.70]), basic computer skill (AOR = 6.74, 95%CI [2.75, 16.56]), and fear of unemployment due to HRIS implementation (AOR = 2.83, 95%CI [1.27–6.32]) were independently associated with readiness of HRIS implementation (Table 5).

**Table 5:** Factors associated with readiness of HRIS implementation of the HR employees in public hospitals and health departments, Amhara Regional state (*N* = 288)

S. no.	Variables		Readiness of HRIS implementation			
			Ready	Not yet ready	COR(95%CI)	AOR(95%CI)
1	Educational status	Diploma and below	48(40.7%)	70(59.3%)	1	1
		First degree+	40(31.2%)	88(68.8%)	0.66(0.39–1.12)	0.69(0.33–1.45)
2	Work experience	Below 5 years	34(45.3%)	41(54.7%)	0.83(0.28–2.44)	0.32(0.07–1.43)
		6–10 years	26(36.1%)	46(63.9%)	0.57(0.19–1.68)	0.35(0.08–1.51)
		11–15 years	13(26.5%)	36(73.5%)	0.36(0.11–1.16)	0.22(0.04–1.05)
		16–20 years	7(20.6%)	27(79.4%)	0.26(0.07–0.94)	0.27(0.05–1.42)
		21 + years	8(50.0%)	8(50.0%)	1	1
3	Availability of personal computer	Yes	17(65.4%)	9(34.6%)	3.96(1.68–9.33)	2.05(0.69–6.06)
		No	71(32.3%)	149(67.7%)	1	1
4	Individual Internet access	Yes	39(58.2%)	28(41.8%)	3.37(2.06–6.64)	2.59(1.19–5.62)*
		No	49(27.4%)	130(72.6%)	1	1
5	Separate room /HR section	Yes	66(53.2%)	58(46.8%)	5.17(2.89–9.25)	8.08(3.69–17.70)*
		No	22(18.0%)	100(82.0%)	1	1
6	Information technology supporter	Yes	49(81.7%)	11(18.3%)	4.37(1.47–13.03)	3.89(.93–16.24)
		No	39(21.0%)	147(79.0%)	1	1
7	Basic computer skill	Yes	76(52.1%)	70(47.9%)	7.96(4.01–15.79)	6.74(2.75–16.56)*
		No	12(12.0%)	88(88.0%)	1	1
8	Fear of unemployment?	Yes	27(31.4%)	59(68.6%)	1	1
		No	61(38.1%)	99(61.9%)	1.35(0.77–2.35)	2.83(1.27–6.32)*

*COR* crude odds ratio, *AOR* adjusted odds ratio, *1* reference

*P value < 0.05

### Challenges of readiness of HRIS implementation

The majority of the respondents included in the in-depth interview mentioned the main challenges related to HRIS implementation readiness were first infrastructure related challenges. These challenges include poor logistics and supply, lack of network and internet, low storage capacity and speed of computers, lack of data backup and anti-virus usage, lack of office equipment, low computer accessories availability, and frequent power interruptions.

The second main challenge raised by the respondents was lack of competency or technical skill-related challenges. These challenges were poor computer skills, lack of competent HR employees (skills, knowledge, and attitude), standard data handling skills, and poor information communication skills.

**Fig. 1:**
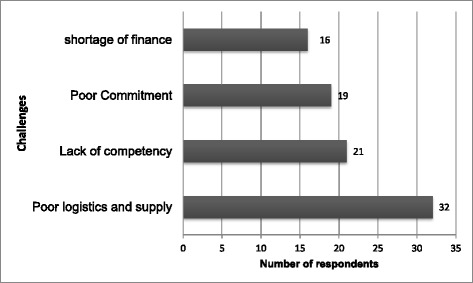
Challenges of readiness of HRIS Implementation

The third challenge explored from the in-depth interviews of this study were related with organizational and managerial challenges such as top management support failure and lack of dedication, failure to work as a team with other departments, low stakeholders’ enthusiasm, low motivation to use information technology, and poor attention to HRIS implementation and HR support process (Fig. 1).

## Discussion

This study aimed to determine the readiness of HRIS implementation, identify associated factors, and explore the challenges of HRIS implementation in hospitals and health departments of the Amhara National Regional State.

In this study, the readiness of HRIS implementation was found to be low. The multivariable logistic regression analysis identified that employees internet access, having separate HR section basic computer skills, and fear of unemployment due to HRIS implementation were found to be significantly associated with the readiness of HRIS implementation.

Employees who had internet and network access are 2.59 times more likely to be ready for the implementation of HRIS. A study in Tanzania also reported non-reliable internet connection as one of the problems in successful implementation of HRIS [12]. Besides this, a survey conducted in Bangladesh and a cross-sectional study from Pakistan indicated lack of information technology support as the main challenge in managing HRIS [9, 30]. However, how the availability of internet in their offices make respondents to be ready for change (in this case, HRIS implementation) needs further exploration.

This study shows that half of the respondents had no separate room for HR section. Employees having separate HR room were eight times more likely to be ready to implement HRIS than those employees who had no separate room. Similarly, a theoretical analysis in Malaysia on adoption of HRIS reported that organizational HR section size was one of the factors for successful adoption of HRIS [31]. This might be due to the reason that having no HR section affects the privacy of employees. A study from Bangladesh reported that lack of privacy was impeding the implementation of HRIS [9].

The current study shows that 40.7% of the respondents had no basic computer skills. Those respondents who had basic computer skills were 6.74 times more likely to be ready to the implementation of HRIS than those having no basic computer skills. A Tanzania study revealed computer skill was improved through training on data utilization (secondary uses of information). In addition, training on data visualization was a necessary factor for successful HRIS implementation. This might be due to the reason that training improves as computer skills among system users which ultimately affects readiness for HRIS implementation [12]. A study conducted in India also indicated that HRIS software training is a key determinant of successful HRIS implementation. This is due to the reason that training enhances HR employees’ skills on the use and installation of user-friendly systems [32].

From all respondents, 35% of them fear that the HRIS will create unemployment and risk their job security. This finding is similar with the study from Bangladesh that shows 40% believed that implementation of HRIS would create an unemployment problem [9]. This might be due to the employees’ perception that if their work is supported by a software, only small number of employees would consequently be required. Those respondents having no fear of unemployment were 2.8 times more likely to be ready for the implementation of HRIS. This may be due to the reason that those employees who do not have unemployment concerns are confident on their skills and ready for change [33]. However, whether having no fear of unemployment is related with HRIS adoption obviously requires further research.

This study also explored shortage of finance, lack of competency, and poor stakeholders’ commitment as challenges of HRIS implementation. Similar to this, a study on HRIS implementation from Jordan shows insufficient capital and skills, shortage of money, and top management support failures and dedication as implementation challenges [13].

Our study has limitations particularly due to social desirability bias that possibly be introduced from respondents of the in-depth interviews. In addition, the respondents of the self-administered questionnaire might have a social desirability bias in self-reporting on the questionnaire. However, we have not observed significant differences between the responses retrieved from observation checklist and from the self-reported questionnaire.

## Conclusion

The readiness of HRIS implementation was found to be low. The availability of internet access, having separate HR section, having basic computer skills, and having no fear of unemployment due to HRIS implementation were positively associated with readiness of HRIS implementation. Poor logistic and supply, lack of competency, poor stakeholders’ commitment, and shortage of finance were the challenges of HRIS implementation. Providing basic computer skills training for HR employees, managers, and executives of hospitals and health departments; providing reliable internet access; and separate HR section would facilitate the successful implementation of HRIS.

## Additional file

Additional file 1: Survey questionnaire. (DOCX 22 kb)

## Abbreviations

eHR: electronic Human Resource
FMOH: Federal Ministry of Health
HIT: Health Information Technician
HR: Human Resource
HRH: Human Resources for Health
HRIS: Human Resource Information System
HRM: Human Resource Management
WHO: World Health organization
