# Supplementary material for: Human Resource Information System implementation readiness in the Ethiopian health sector: a cross-sectional study
Source: Hum Resour Health. 2017 Dec 20;15:85. doi: 10.1186/s12960-017-0259-3 (PMC5738912; doi:10.1186/s12960-017-0259-3)
Supplement: Additional file 1: — Survey questionnaire. (DOCX 22 kb) [file 12960_2017_259_MOESM1_ESM.docx]

## Questionnaire

The data collection instrument, English version.

College of medicine and Health Sciences, Institute of public health, University of Gondar.

A Questionnaire for assessment of Readiness of HRIS implementation and associated factors in hospitals and health departments of the Amhara Regional State, Ethiopia, 2016.

**PART 1: SOCIO-DEMOGRAPHIC CHARACTERISTICS**

***Direction: Choose your answer and circle/tick✔/ it***

|  | **Variables** | **Responses** | **Remarks** |
| --- | --- | --- | --- |
| 1 | Sex | 1. Male 2. Female |  |
| 2 | Age | __________ |  |
| 3 | Marital Status | 1. Single 3. Married  2. Divorced 4. Widowed |  |
| 4 | Religion | 1. Orthodox 3. Muslim 2. Protestant4.Others |  |
| 5 | Educational Status | 1. Below college diploma  2. College Diploma  3. First Degree  4. 2^nd^ Degree and above |  |
| 6 | Program enrolled | 1. Regular 2. Distance 3. Extension 4. Upgrading/in-*service training* 5. Other specify_______­______ |  |
| 7 | Position | 1. Data Clerk  2. HR Manager/head  3. HR Case Worker/ employees  4. HIT  5. Other (Specify)_________ |  |
| 8 | Work Experience | ­­­_________years |  |
| 9 | Salary | _________Birr |  |

Part 2: Organizational factors

| 2.1 | Type of health Organization you are working?*(select only one)* | | | 🞏 Zonal Health Department | | 🞏 Referral  Hospital | | | | 🞏 Teaching & Referral Hospital | | |
| --- | --- | --- | --- | --- | --- | --- | --- | --- | --- | --- | --- | --- |
|  |  |  |  | 🞏Primary  Hospital | | 🞏 Town Administration Health Department Referral | | | | | | |
| # | Questions | | | | | | Yes | | No | | Remark | |
| 2.2 | Do you have a telephone in HR office or access to a telephone? | | | | | | 🞏 | 🞏 | | | |  |
| 2.3 | Do you have electrical power outlets in HR section/ work area? | | | | | | 🞏 | 🞏 | | | |  |
| 2.4 | How many computers are there in HR Section immediate working group? | | | | | | Number:________ | | | | | |
| 2.5 | Does HR section have access to own computer or a shared computer? | | | | | | 🞏 | 🞏 | | | |  |
| 2.6 | Do you have network access via this computer? | | | | | | 🞏 | 🞏 | | | |  |
| 2.7 | Do you have Internet access via this computer? | | | | | | 🞏 | 🞏 | | | |  |
| 2.8 | Do you have Functional Printer? | | | | | | 🞏 | 🞏 | | | |  |
| 2.9 | Do you have Backup power supply to run computers? | | | | | | 🞏 | 🞏 | | | |  |
| 2.10 | Is there separate room for HR section? | | | | | | 🞏 | 🞏 | | | |  |
| 2.11 | Is there separate budget allocation for HR section to implement HRIS? | | | | | | 🞏 | 🞏 | | | |  |
| 2.12 | Do you have HRIS policy manual available? | | | | | | 🞏 | 🞏 | | | |  |
| 2.13 | Do you have HR indicators used for data collection purpose? | | | | | | 🞏 | 🞏 | | | |  |
| 2.14 | Is there someone in your organization who is qualified to keep the computer(s) functioning well being routinely available to deal with any issues? | | | | | | 🞏 | 🞏 | | | |  |
| 2.15 | If your answer for Q2.14 is “**Yes”**, is this person located on-site? | | | | | | 🞏 | 🞏 | | | |  |
| 2.15 | If your answer for Q2.14 **“No**”, how often are any other available? | | | | | | | | | | | |
|  | 🞏 On call | 🞏 Daily | 🞏 Weekly | | 🞏 Monthly | | 🞏 Other (specify): | | | | | |

Part 3: Technical factors

| # | Questions | Yes | No | Remark | |
| --- | --- | --- | --- | --- | --- |
| 3.1 | Do you think that there is a developed information technology infrastructure (i.e., hardware, software, networks), and human expertise? | 🞏 | 🞏 |  |  |
| 3.2 | Is your computer comfortable to use? | 🞏 | 🞏 |  |  |
| 3.3 | If “**NO**” for Question 3.2 list down the problems______________________________ __________________________________________________________________ | | | |  |
| 3.4 | Do you use anti-virus for your computer? | 🞏 | 🞏 |  |  |
| 3.5 | Is HRIS software user friendly? | 🞏 | 🞏 |  |  |
| 3.6 | If “**NO**” for Question 3.5 list down cases_____________________________________ ________________________________________________________________________________________________________________________________________ | | | |  |
| 3.7 | Are modules of HRIS compatible with the HR workflow? | 🞏 | 🞏 |  |  |

Part 4: Personal Factors

| # | Questions | Yes | No |  |
| --- | --- | --- | --- | --- |
| 4.1 | Knowledge, skill and Attitude of HRIS   - - 1. Do you know the modules of HRIS? (list)     2. Do you know Advantages of HRIS? (list)     3. Do you have basic computer skill?     4. Can you install HRIS software?     5. Can you configure the software?     6. Can you manipulate all the modules?     7. Do you believe that HRIS is important for your organization?     8. Do you think that you have roles in implementation of HRIS? | 🞏  🞏  🞏  🞏  🞏  🞏  🞏  🞏 | 🞏  🞏  🞏  🞏  🞏  🞏  🞏  🞏 |  |
| 4.2 | Did you receive training on your HRIS with its policies? | 🞏 | 🞏 |  |
| 4.3 | Is there a manual or handbook on HRIS with its policies? | 🞏 | 🞏 |  |
| 4.4 | Is there a starting of the HRIS and/or policies for all employees in your department? | 🞏 | 🞏 |  |
| 4.5 | Do you think that HRIS will create unemployment? | 🞏 | 🞏 |  |
| 4.6 | Do you feel that you are responsible for full implementation of the system? | 🞏 | 🞏 |  |
| 4.7 | Can HRIS enhance efficiency of HRM in the organization? | 🞏 | 🞏 |  |
| 4.8 | Can HRIS software be fully applicable in your organization? | 🞏 | 🞏 |  |

PART 5:In-depth interview Questions for Medical Directors, Managers and head of health Departments.

| 5.1 | Do have the information about HRIS before? Have you taken training about HRIS? |
| --- | --- |
| 5.2 | In your opinion what are the advantages HRIS for your Organization? |
| 5.3 | In your assumption if HRIS is implemented what challenges and limitations may encounter your organization to be successful? |
| 5.4 | What changes would you recommend be made to implement the system?  About:   1. HR section employee’s competency? 2. How is ICT organized? 3. How is executives support and commitment? 4. How training has to be done? |
| 5.5 | Would you please provide other information about the system that could help the HRIS implementation? |

**Observation Check list**

| # | Questions | Yes | No | Remark |
| --- | --- | --- | --- | --- |
| 1 | Availability of Electric Generator for the organization /Facility | 🞏 | 🞏 |  |
| 2 | Availability separate section for HR core process | 🞏 | 🞏 |  |
| 3 | Fulfillment of necessary Ergonomics of the HR core process | 🞏 | 🞏 |  |
| 4 | Is there necessary electric installation? | 🞏 | 🞏 |  |
| 5 | Did the workers have their own Identified work place? | 🞏 | 🞏 |  |
| 6 | Availability of Necessary Version (2010) Computes? | 🞏 | 🞏 |  |
| 7 | Availability Computer Accessories? | 🞏 | 🞏 |  |
